# Nucleotide diversity and population differentiation of the Melanocortin 1 Receptor gene, *MC1R*

**DOI:** 10.1186/1471-2156-9-31

**Published:** 2008-04-10

**Authors:** Sharon A Savage, Meg R Gerstenblith, Alisa M Goldstein, Lisa Mirabello, Maria Concetta Fargnoli, Ketty Peris, Maria Teresa Landi

**Affiliations:** 1 Clinical Genetics Branch, Division of Cancer Epidemiology and Genetics, National Cancer Institute, National Institutes of Health, Department of Health and Human Services, USA; 2 Genetic Epidemiology Branch, Division of Cancer Epidemiology and Genetics, National Cancer Institute, National Institutes of Health, Department of Health and Human Services, USA; 3 Department of Dermatology, University of L'Aquila, Via Vetoio – Coppito 2, 67100 L'Aquila, Italy

## Abstract

**Background:**

The melanocortin 1 receptor gene (*MC1R*) is responsible for normal pigment variation in humans and is highly polymorphic with numerous population-specific alleles. Some *MC1R *variants have been associated with skin cancer risk.

**Results:**

Allele frequency data were compiled on 55 single nucleotide polymorphisms from seven geographically distinct human populations (n = 2306 individuals). *MC1R *nucleotide diversity, π, was much higher (10.1 × 10^-4^) than in other genes for all subjects. A large degree of population differentiation, determined by F_ST_, was also present, particularly between Asia and all other populations, due to the p.R163Q (c.488 G>A) polymorphism. The least amount of differentiation was between the United States, Northern Europe, and Southern Europe. Tajima's D statistic suggested the presence of positive selection in individuals from Europe.

**Conclusion:**

This study further quantifies the degree of population-specific genetic variation and suggests that positive selection may be present in European populations in *MC1R*.

## Background

Differences in human skin pigmentation have been attributed to genetic variation in several different genes [1-3]. Among these, the melanocortin 1 receptor gene (*MC1R*, MIM#155555), a member of the G protein-coupled receptors superfamily, is the major contributor to normal pigment variation in humans. It is a small, highly polymorphic gene consisting of one exon with 951 coding nucleotides on chromosome 16q24.3.

Numerous studies have demonstrated associations between specific *MC1R *variants and red hair, light skin, poor tanning ability and heavy freckling [4-9]. A recent genome-wide association scan confirmed the role of *MC1R *SNPs in hair, eye, and skin pigmentation[3]. The functional role of many of these variants has been described [10-13]. Several *MC1R *variants are also associated with increased risk of malignant melanoma in a variety of populations [14-22] The effect of *MC1R *polymorphisms in melanoma risk appears to extend beyond its effect on pigmentation in most of these investigations, and to be linked to melanomas harboring mutations in the *BRAF *oncogene[23].

Several hypotheses have been generated in an effort to understand the evolutionary history of skin pigmentation in humans. It has been suggested that as humans migrated out of Africa to climates with more limited exposure to sunlight, relaxation of functional constraints in pigmentation genes, including *MC1R*, or selection for functionally relevant variants that led to lighter skin pigmentation occurred[24]. This could result in an improved ability to synthesize vitamin D in the presence of limited sunlight exposures [25-27]. It has also been suggested that darker skin is favored in regions closer to the equator for protection against ultraviolet radiation[24]. In addition, differences in skin pigmentation could protect against pathogens and cold injury, and may have also been important in sexual selection[28].

Genetic variation of *MC1R*, in the form of single nucleotide polymorphisms (SNPs), is significantly different across populations from different geographic regions [29,30]. In most regions of the genome, there is a higher degree of genetic variation in individuals of African descent than in other populations, most likely due to evolutionary history [31,32]. *MC1R *is an exception to this observation. It has been shown to be more polymorphic in individuals of European descent than in those from Africa [29,30]. A comprehensive study of SNP allele frequencies in *MC1R *from populations around the world, further quantified the large differences in the distribution of variants across populations, with a prominent difference between light and dark-pigmented individuals [29]. The goal of the current study was to expand on that study of *MC1R *genetic variation by characterizing nucleotide diversity, population specific differentiation (F_ST_), and to study measures of selection.

## Results

### Nucleotide Diversity

Allele frequency (AF) data was compiled from a total of 2306 individuals who were grouped into seven populations, based on geographic location (Table 1). The actual number of individuals in each group was Africa 117, India 53, Southern Europe 838, Northern Europe 650, Asia 343, Papua New Guinea 40, and the United States 265. The number of SNPs per population evaluated in the study ranged from three (India) to 36 (Southern Europe), with a total of 55 SNPs studied in *MC1R *in these analyses. AFs are shown in Table 2. Thirty-seven SNPs were nonsynonymous (NS) and 18 were synonymous (S). The greatest number of SNPs was present in individuals from Southern Europe (29 NS, 7 S) and Northern Europe (18 NS and 3 S). The fewest number of SNPs were noted in the subjects from India (2 NS, 1 S), Papua New Guinea (2 NS, 2 S) and Africa (3 NS and 8 S).

**Table 1:** Description of populations and studies used for analyses of *MC1R *allele frequencies

**Population Name**	**Description**	**# Individuals**	**Reference**
Africa	Healthy subjects from South Africa (22 Negroid and 17 San people), 25 from South Africa, Nigeria, Zaire, Kenya and Gambia, and 53 from The Ivory Coast and Gambia	117	John 2003, Rana 1999, Harding 2000
India	Healthy subjects from studies investigating MC1R variants.	53	Rana 1999, Harding 2000
Southern Europe	Healthy subjects from Spain (188), Italy (495) and Greece (155)	838	Fernandez 2007, Harding2000, Fargnoli 2003, Pastorino 2004, Landi 2005, Fargnoli 2006, Stratigos 2006,
Northern Europe	Healthy subjects from The Netherlands (385), Britain/Ireland (93) and France (172)	650	Bastiaens 2001, Kennedy 2001, Flanagan 2000, Harding 2000, Chaudru 2005
Asia	Healthy subjects from East and Southeast Asia. Rana et al reported individuals from China (50), Japan (4), Cambodia (1), Vietnam (1), Siberian Yakut (5) and Mongolia (4). The remaining studies were of individuals from Japan.	343	Rana 1999, Harding 2000, Motokawa 2006, Nakayama 2006
Papua New Guinea	Healthy subjects from studies investigating MC1R variants.	40	Harding 2000, Nakayama 2006
United States	Healthy Caucasian subjects participating in a population based study conducted in Philadelphia, PA and Caucasian spouse controls from the US in a familial melanoma case/control study.	265	Kanetsky 2004, Goldstein 2005

**Table 2:** Allele frequencies (%) of *MC1R *single nucleotide polymorphisms in seven populations.

**protein**	**nucleotide**	**All subjects**	**Africa**	**Asia**	**India**	**NoEur**	**SoEur**	**US**	**PNG**
p.P18A	c.52 C>G	0	0	0	0	0.08	0	0	0
p.C35Y	c.104 G>A	0	0	0	0	0	0.12	0	0
p.V38M	c.112 G>A	0	0	0	0	0	0.06	0	0
p.L44V	c.130 C>G	0	0	0	0	0	0.06	0	0
p.F45L	c.133 T>C	0	0	0	0	0	0.24	0	0
p.S47I	c.140 G>T	0	0.43	0	0	0	0	0	0
p.L50L	c.150 G>A	0	1.28	0	0	0	0	0	0
p.V60L	c.178 G>T	0.10	0	0	0	10.69	15.75	13.21	0
p.R67Q	c.200 G>A	0	0	1.17	0	0	0.06	0	0
p.A81P	c.241 G>C	0	0	0	0	0.08	0	0	0
p.S83P	c.247 T>C	0	0	0	0	0.15	0.12	0.19	0
p.S83L	c.248 C>T	0	0	0	0	0	0.06	0	0
p.D84E	c.252 C>A	0	0	0	0	1.00	0.12	1.51	0
p.V92M	c.274 G>A	0.07	0	13.41	0.94	8.00	3.70	9.06	3.75
p.T95M	c.284 C>T	0	0	0	0	0.08	0.12	0	0
p.L99I	c.295 C>A	0	0.43	0	0	0	0	0	0
p.A103A	c.309 C>T	0	0.43	0	0	0	0	0	0
p.L106Q	c.317 T>A	0	0	0	0	0	0.06	0	0
L106L	c.318 G>A	0	1.71	0	0	0	0.12	0	0
p.A111V	c.332 C>T	0	0	0	0	0	0.18	0	0
p.I120T	c.359 T>C	0	0	1.75	0	0	0.06	0	0
p.V122M	c.364 G>A	0	0	0	0	0	0.24	0	0
p.V122V	c.366 G>C	0	0	0	0	0	0.12	0	0
p.R142H	c.425 G>A	0.01	0	0	0	0.62	1.31	0.75	0
p.R151C	c. 451 C>T	0.03	0	0	0	5.62	3.16	6.42	0
p.Y152X	c.456 C>A	0	0	0	0	0	0.06	0	0
p.I155T	c.464 T>C	0	0	0	0	0.23	0.48	1.51	0
p.R160W	c.478 C>T	0.04	0	0	0	8.31	1.85	7.17	0
p.R160Q	c.479 G>A	0	0	0	0	0	0.06	0	5
p.R163Q	c.488 G>A	0.14	0	75.51	4.72	4.62	1.61	4.15	0
p.A166A	c.498 G>A	0	0	0	0	0	0	0	11.25
p.I168I	c.504 C>T	0	0.43	0	0	0	0	0	0
p.A171D	c.512 C>A	0	0	0	0	0	0.06	0	0
p.V174I	c.520 G>A	0	0	0	0	0.08	0	0	0
p.Y182Y	c.546 C>T	0	0	0	0	0	0	0.19	0
p.F196L	c.586 T>C	0	2.14	0.15	0	0.08	0	0	0
p.R213W	c.637 C>T	0	0	0	0	0	0.06	0	0
p.G220G	c.660 C>T	0	0	0	0	0	0.06	0	0
p.Q228Q	c.684 G>A	0	0	0	0	0	0.12	0	0
p.P230L	c.689 C>T	0	0	0	0	0.31	0	0	0
p.P230P	c.690 G>A	0	0	0	0	0.08	0	0	0
p.Q233Q	c.699 G>A	0	0	0	0	0	0.36	0.19	0
p.A240A	c.720 T>C	0	0	0	0	0	0	0.19	0
p.T242T	c.726 C>T	0	0	0	0	0	0.12	0	0
p.G248V	c.743 G>T	0	0	0	0	0	0.06	0	0
p.H260P	c.779 A>C	0	0	0	0	0.23	0	0	0
p.I264I	c.792 C>T	0	0	0	0	0.15	0	0	0
p.V265V	c.795 C>G	0	1.28	0	0	0	0	0	0
p.C273C	c.819 C>T	0	0.43	0	0	0	0	0	0
p.K278E	c.832 A>G	0	0	0	0	0.23	0.24	0	0
p.N279K	c.837 C>A	0	0	0	0	0	0.06	0	0
p.D294H	c.880 G>C	0.01	0	0	0	1	1.43	2.45	0
p.F300F	c.900 C>T	0	2.99	0	0	0	0	0	0
p.T308M	c.923 C>T	0	0	0	0	0	0.06	0	0
p.T314T	c.942 A>G	0.09	44.44	13.27	13.21	7.69	2.33	10.75	18.75

Abbreviations: SoEur, Southern Europe, NoEur, Northern Europe, PNG, Papua New Guinea, US, United States. Allele frequency % were rounded to two decimal places, therefore, rare alleles may be reported as zero due to rounding.

In order to correct for the variable population sizes, which could contribute to the absolute number of SNPs identified, π and θ, measures of nucleotide diversity, were calculated. The overall nucleotide diversity for all SNPs in *MC1R*, as measured by π, was 10.1 × 10^-4 ^for all populations (2306 total subjects); it ranged from a low of 3.6 × 10^-4 ^in subjects from India to a high of 11.1 × 10^-4 ^from US subjects (Table 3). In subgroup analyses of the Northern and Southern European populations, π was the highest in subjects from Britain (18.1 × 10^-4^). θ, the population mutation parameter, was quite variable across populations, ranging from 6 × 10^-4 ^in India to 47.3 × 10^-4 ^in Southern Europe. In Southern European subjects, θ was the highest, 43.6 × 10^-4^, in Italy.

**Table 3:** *MC1R *nucleotide diversity, Tajima's D, and Fu's F_S _statistic for seven populations Statistically significant values are bolded.

			π × 10^-4^		θ × 10^-4^					
	# SNPs	# NS SNPs	All SNPs	NS only	All SNPs	NS only	Tajima's D statistic	Tajima's D p-value	Fu's F_S_	Fu's F_S _p-value

**Africa**	11	3	7.6	0.6	19.2	5.2	**-1.41**	**0.048**	-0.24	0.436
**India**	3	2	3.6	1.2	6.0	4.0	-0.72	0.27	-1.27	0.202
**SoEur**	36	29	6.7	6.0	47.3	38.1	**-2.13**	**<0.001**	**-27.92**	**<0.001**
**Greece**	12	10	5.3	4.2	20.0	16.7	**-1.71**	**0.008**	-3.81	0.071
**Italy**	31	26	7.1	6.8	43.6	36.6	**-2.10**	**<0.001**	-6.46	0.055
**Spain**	17	15	6.7	5.4	27.5	24.2	**-1.85**	**0.003**	**-8.44**	**0.009**
**NoEur**	21	18	9.6	8.1	28.5	24.4	**-1.53**	**0.026**	**-23.66**	**<0.001**
**Britain**	10	10	18.1	10.9	10.9	18.1	-0.94	0.181	-2.07	0.207
**France**	12	10	9.4	7.4	19.7	16.4	-1.20	0.099	-2.92	0.131
**Netherlands**	16	14	9.3	7.6	23.3	20.4	**-1.36**	**0.047**	-4.74	0.095
**US**	14	10	11.1	9.0	21.5	15.4	-1.10	0.126	-2.41	0.212
**Asia**	6	5	9.4	7.0	8.9	7.4	1.05	0.855	2.15	0.839
**PNG**	4	2	7.2	1.8	8.5	4.2	-0.33	0.433	-0.78	0.301

Abbreviations: SNPs, single nucleotide polymorphisms; NS, nonsynonymous; SoEur, Southern Europe; NoEur, Northern Europe; PNG, Papua New Guinea; US, United States

Nucleotide diversity was also calculated for NS SNPs only to further understand the contribution of these SNPs to *MC1R *genetic variation (Table 3). π in NS SNPs ranged from a low of 0.6 × 10^-4 ^in subjects from Africa to a high of 9.0 × 10^-4 ^in the US. Within the European populations, π for NS SNPs was the greatest in Britain (10.9 × 10^-4^). As was seen for all SNPs, θ for NS SNPs was the highest in Italy (36.6 × 10^-4^).

In addition to a high degree of inter-population variability, *MC1R *has a higher degree of nucleotide diversity in comparison to other groups of genes (Table 4). Studies of genetic variation in various gene groups, e.g., genes important in telomere biology [33], antigen processing and presenting genes [34], pharmaceutical response [35], and environmental response genes [36], showed π values ranging from 3.0 × 10^-4 ^to 6.7 × 10^-4^, while all seven populations studied for *MC1R *had a combined π value of 10.1 × 10^-4^. θ showed similarly higher values in *MC1R *(64.2 × 10^-4^) than in other sets of genes (all <8.4 × 10^-4^).

**Table 4:** Comparison of *MC1R *nucleotide diversity and Tajima's D statistic to other sets of genes. These data include subjects from different ethnic groups calculated as a whole. The number of genes evaluated is shown in parentheses.

	π × 10^-4^	θ × 10^-4^	# NS/kbp	#S/kbp	Tajima's D statistic
*MC1R*	10.1	64.2	38.9	18.9	-2.08
Telomere biology genes^a ^(n = 7)	3.0	5.5	0.59	1.01	-1.365
Nuclear hormone receptor genes^b ^(n = 40)	4.1	7.5	1.55	1.97	ND
Antigen processing and presenting genes^b ^(n = 72)	4.7	8.3	2.29	1.99	ND
Reference genes^b ^(n = 4950)	4.8	7.9	1.88	1.86	ND
Pharmaceutical Response genes^c ^(n = 1598)	5.6	ND	ND	ND	negative
Environmental response genes^d ^(n = 213)	6.7	ND	ND	ND	ND

^a^Savage et al. [2005].

^b^Pungliya et al. [2004].

^c^Schneider et al. [2003].

^d^Livingston et al. [2004].

Abbreviations: NS, nonsynonymous, S, synonymous, ND, not determined in the reference manuscript.

### Population Differentiation

The F_ST _statistic, a pairwise measure of population differentiation, has been used extensively to compare the degrees of heterozygosity across populations[2,37]. Therefore, *MC1R *F_ST _was calculated for each of the described populations (Table 5). Overall, a very high degree of differentiation was noted between Asia and each of the other groups; F_ST _ranged from 0.459 between Asia and Africa to 0.356 between Asia and the United States.

**Table 5:** F_ST _statistic for *MC1R *in seven populations.

	**India**	**SoEur**	**NoEur**	**Asia**	**PNG**	**US**
**Africa**	0.157	0.232	0.168	0.459	0.101	0.145
**India**		0.073	0.042	0.455	0.029	0.044
**SoEur**			0.016	0.437	0.089	0.017
**NoEur**				0.369	0.057	0.001
**Asia**					0.430	0.356
**PNG**						0.053

Abbreviations: SoEur, Southern Europe, NoEur, Northern Europe, PNG, Papua New Guinea, US, United States.

The degree of differentiation between subjects from Africa and the six other groups ranged from 0.101 (Papua New Guinea) to 0.232 (Southern Europe). Papua New Guinea had relatively modest degrees of population differentiation when compared to all populations except Asia, where it was very large. The least amount of population differentiation was found in comparisons between the United States, Northern Europe, and Southern Europe.

We also performed analyses on the subpopulations that comprised the Northern Europe (Britain, the Netherlands, and France) and the Southern Europe (Greece, Italy, and Spain) groups. F_ST _values for all of these comparisons were between 0 and 0.03, suggestive of little differentiation (data not shown).

### Selection

Several studies have identified signals of positive selection in pigmentation genes in subjects from East Asia, and Europe but not from those in Africa[2,38,39]. Whether or not positive selection is present at the *MC1R *locus, is an area of active investigation. Previous work suggested that the high degree of variation in *MC1R *is not due to selection but rather to a relaxation of functional constraint outside of Africa [25]. In order to further test this hypothesis we first determined Tajima's D statistic, a measure of the relationship between the number of segregating sites (SNPs) and nucleotide diversity (Table 3). It was not statistically significant (p > 0.05) in the populations from India, Asia, Papua New Guinea, and the United States, in which Tajima's D values were -0.72, 1.05, -0.33, and -1.10, respectively. The African population studied had Tajima's D value of -1.41 and a p-value of 0.048. Statistically significant and negative Tajima's D values were present in the Southern European population (-2.13, p = <0.001). Subgroup analyses of this population showed the same trend, with negative Tajima's D values and p-values <0.05 in Greece, Italy, and Spain. The combined Northern European group had a Tajima's D value of -1.53 (p = 0.026). However, only the population from the Netherlands had a statistically significant Tajima's D value in this group (-1.36, p = 0.047).

The F_S _test of neutrality developed by Fu (1997) and based on θ is a powerful method to further evaluate the polymorphic patterns under population growth and genetic hitchhiking. These values are shown in Table 3. Fu's F_S _was statistically significant in the grouped Southern European population (-27.92, p = <0.001) but only in the subpopulation from Spain (-8.44, p = 0.009). The Northern European population group also had a statistically significant F_S _value (-23.66, p = <0.001). The F_S _values were not statistically significant in the other populations. Fu and Li's D values were comparable in scope to Tajima's D in this study but p-values were not obtainable due to software limitations (data not shown).

## Discussion

*MC1R *is a small, highly variant gene. This study evaluated the nucleotide diversity, population-specific differentiation, and tested for positive selection of *MC1R *based on a compilation of previously published data. We used allele frequencies from studies that reported sequencing the entire gene and used the 951 coding bp of *MC1R *as the reference sequence. The genotype data used for these analyses was derived from the sample size and reported AFs, as a result, we were unable to assess the haplotype structure of *MC1R*. Also, we could not assess the flanking sequences of *MC1R *because the exact regions sequenced were not reported in the published data.

We observed that nucleotide diversity, as measured by π and θ, was greater in *MC1R *than in other groups of genes. It should be noted that these differences may be somewhat skewed because our study evaluated only one, small gene, *MC1R*, while the other studies evaluated between 7 and 4950 different genes [33-36]. Several other studies have noted the highly polymorphic nature of *MC1R *(compiled by Gerstenblith *et al*. [29]). Overall, the largest degree of nucleotide diversity was seen between Asia and all other populations, most likely due to the presence of the R163Q (c.488 G>A) SNP, which was present in 75% of Asians studied, *versus *less than 5% of any of the other six populations. It has been suggested that this allele may be present due to a bottleneck in Asian demographic history [25]. This allele has been shown to have reduced cell surface expression with corresponding impairment in cAMP coupling and effects in pigmentation [13].

The F_ST _statistic was calculated to further assess the degree of population-specific differentiation in *MC1R*. Numerous population-specific SNPs and an overall high degree of population differentiation, as measured by F_ST _between populations, are present in *MC1R*, particularly in Asians. Minimal differentiation was noted between Southern Europe, Northern Europe, and the United States. These individuals were identified in previous studies as Caucasian (i.e. of European descent), and likely share some degree of common ancestry. Although frequency of specific variants differ across populations of European descent (e.g. the allele frequency of the T allele of c.451C>T, p.R151C, was 1.9% in Greece but 10.2% in Britain/Ireland[29]), the sub-group analysis of the Northern and Southern European groups showed little evidence of population differentiation. This is consistent with other studies showing little among-population differentiation[25,27,30]. The African population in this study had moderate to large degrees of differentiation in comparison to other populations. This is consistent with prior *MC1R *SNP data showing fewer variants in individuals from Africa when compared to non-African populations[29].

Tajima's D statistic tests whether or not a gene or genomic region is evolving randomly (neutral evolution) or if the region is under selection (non-neutral evolution). It is based on the spectrum of AFs at different sites, as well as on population size. Tajima's D statistic was used to test *MC1R *for the presence of selection. These data, which are based on the very large sample size described herein, suggest that positive selection may be present in the Southern European population as a whole, as well as in its three subgroups, Greece, Italy, and Spain, based on the presence of negative Tajima D values and statistically significant p-values. The data also suggest the presence of some degree of positive selection in the Northern European population; but only the subgroup from the Netherlands had statistically significant p-values. It should be noted that Tajima's D statistic assumes that all nucleotides are equally mutable, subject to the same population dynamics, and can be misleading if a significant population bottleneck occurred. We also used Fu's F_S _test to address the presence of positive selection versus neutral evolution of *MC1R*. This was statistically significant only in the Southern and Northern European groups and the subpopulation from Spain and further suggests the presence of positive selection in the European populations. However, our data are also limited because we were only able to study the 951 bp of coding sequence in *MC1R *and were not able to assess the larger genomic region.

Several studies have evaluated genetic adaptation of the *MC1R *gene for evidence of positive selection with conflicting results. Some studies suggested that purifying selection is present in Africa and that relaxation of functional constraint in non-African populations, instead of positive selection, is present[25,27,40]. On the other hand, most recent studies have found evidence of positive selection at other pigmentation genes. For example, Myles *et al *[2] found evidence for positive selection in the *DCT *gene among individuals of Chinese ancestry. In their study, *MC1R *interpretations were limited because of the different SNPs genotyped between the Perlegen and HapMap data sets studied. In a study of 118 putative skin pigmentation genes, data were consistent with positive selection in subjects from Europe (*OCA2, TYRP1*, and *KITLG*) and in Asians (*DCT, EGFR*, and *DRD2*)[38]. Unfortunately, *MC1R *could not be evaluated in that study due to ascertainment criteria. It was also suggested that at least weak, recent positive selection may be present in *MC1R*, based on the AF variability between CEPH Utah and East Asian HapMap samples[3]. Our data suggest that *MC1R *may be under positive selection in some populations, although additional studies are needed to further evaluate this finding.

## Conclusion

This study further quantifies the degree of *MC1R *genetic variation, illustrates the complexity of this variation across numerous populations, and suggests that positive selection plays a role in European populations. Understanding of population-specific genetic variation in *MC1R *and the role it plays not just in skin pigmentation, but sun sensitivity and melanoma risk, has the potential to impact clinical care and public health.

## Methods

AF data of *MC1R *SNPs from populations around the world were compiled as described [29], from twenty-two skin cancer case-control and population studies that fully sequenced *MC1R *in distinct populations. From the studies included in Gerstenblith *et al *[29], we restricted our analyses to those that included data from healthy, control individuals [7,8,16,18,19,21,22,25,27,40-47]. We excluded studies that noted only the presence of a SNP but not actual AFs [4,48,49], that measured AFs on family members [4,9,18], that were restricted to the extremes of hair and skin color phenotypes [6], or to ethnically diverse groups in which it was not possible to determine the AFs for each ethnic group [17]. In addition, the study of *MC1R *variants in individuals from Spain was also included [22]. Populations from Europe and the United States were identified as Caucasian individuals in these studies. European populations were combined based on geographic locations. Northern Europe consisted on subjects from Britain, France, and the Netherlands. Southern Europe consisted of subjects from Greece, Italy, and Spain. The populations and studies from which they were derived are shown in Table 1.

Genotype data files were created from AFs for each population (Table 2) and were then analyzed in DNAsp version 4.0 [50]. The 951 base pairs (bp) of coding *MC1R *sequence (NM_002386) was used as the template. Since data files were created from AFs, we were unable to assess the haplotype structure of *MC1R*. Analyses were performed that were not dependent on haplotype structure. Nucleotide diversity (π), the average number of nucleotide substitutions per site between two sequences, was calculated with the Jukes and Cantor correction [51]. Theta (θ), the population mutation parameter (two times the mutation rate per site per generation times the number of heritable units in the population), was calculated on a base pair basis from the total number of segregating sites (SNPs) under the no recombination model [52]. Genetic differentiation among populations was measured by F_ST_[53]. Arlequin (v3.11) was used to determine Tajima's D statistic and Fu's F_S _test[54] under a neutral model with 1000 simulations [55,56]. Other genes and corresponding estimates of population differentiation were selected for comparison with *MC1R *values [33-36].

## Authors' contributions

SAS participated in the study design, performed all the population genetics analyses, data interpretation, and wrote the manuscript. MRG reviewed all manuscripts related to *MC1R*, analyzed the *MC1R *frequency in each population, summarized all data for the population genetics analyses, and participated in data analysis and interpretation. AMG participated in the study design and oversaw statistical analyses and data interpretation; LM participated in data analysis and interpretation. MCF and KP participated in the study design and data interpretation. MTL was responsible for the conception and design of the study, and oversaw data analysis and interpretation. All authors contributed to the manuscript's writing and review.
